# Histone H3 Acetyl K9 and Histone H3 Tri Methyl K4 as Prognostic Markers for Patients with Cervical Cancer

**DOI:** 10.3390/ijms18030477

**Published:** 2017-02-23

**Authors:** Susanne Beyer, Junyan Zhu, Doris Mayr, Christina Kuhn, Sandra Schulze, Simone Hofmann, Christian Dannecker, Udo Jeschke, Bernd P. Kost

**Affiliations:** 1 Department of Obstetrics and Gynecology, Ludwig-Maximilians-University of Munich, 80337 Munich, Germany; sannebeyer@yahoo.de (S.B.); Junyan.Zhu@med.uni-muenchen.de (J.Z.); christina.kuhn@med.uni-muenchen.de (C.K.); Sandra.schulze@med.uni-muenchen.de (S.S.); simone.hofmann@med.uni-muenchen.de (S.H.); Christian.dannecker@med.uni-muenchen.de (C.D.); bernd.kost@med.uni-muenchen.de (B.P.K.); 2 Department of Pathology, Ludwig-Maximilians-University of Munich, 80337 Munich, Germany; doris.mayr@med.uni-muenchen.de

**Keywords:** cervical cancer, histone H3 acetyl K9, histone H3 tri methyl K4, epigenetics, chromatin modification, histone proteins, prognosis

## Abstract

Chromatin remodeling alters gene expression in carcinoma tissue. Although cervical cancer is the fourth most common cancer in women worldwide, a systematic study about the prognostic value of specific changes in the chromatin structure, such as histone acetylation or histone methylation, is missing. In this study, the expression of histone H3 acetyl K9, which is known to denote active regions at enhancers and promoters, and histone H3 tri methyl K4, which preferentially identifies active gene promoters, were examined as both show high metastatic potential. A panel of patients with cervical cancer was selected and the importance of the histone modifications concerning survival-time (overall survival and relapse-free survival) was analyzed in 250 cases. Histone H3 acetyl K9 staining was correlated with low grading, low FIGO (TNM classification and the International Federation of Gynecology and Obstetrics) status, negative N-status and low T-status in cervical cancer, showing a higher expression in adenocarcinoma than in squamous cell carcinoma. Cytoplasmic expression of histone H3 tri methyl K4 in a cervical cancer specimen was correlated with advanced T-status and poor prognosis. While cytoplasmic H3K4me3 expression seemed to be a marker of relapse-free survival, nuclear expression showed a correlation to poor prognosis in overall survival. Within this study, we analyzed the chemical modification of two histone proteins that are connected to active gene expression. Histone H3 acetyl K9 was found to be an independent marker of overall survival. Histone H3 tri methyl K4 was correlated with poor prognosis and it was found to be an independent marker of relapse-free survival. Therefore, we could show that chromatin remodeling plays an important role in cervical cancer biology.

## 1. Introduction

Cervical cancer is the fourth most frequent cancer in women worldwide (about 530,000 new cases in 2012, 7.5% of all female cancer deaths). The leading cause of cervical cancer is a persistent infection with high-risk human papillomavirus (HR-HPV) [1]. Specifically, the HPV subtypes 16 and 18 cause about 70% of all cancer cases [1,2]. A total of 170 HPV-types have been described currently [3]. The infection with 15 types of HPV most likely leads to cancer, which is why these 15 types are called carcinogenic or high-risk types [4]. The genome of human papillomaviruses consists of approximately 8000 base pairs and contains six “early genes” (E6, E7, E1, E2, E4, E5) and two “late genes” (L1, L2) [5]. In case of replication of the viral gene E6, the E6 oncoprotein is expressed, which disturbs the cell cycle [6]. E6 oncoprotein and E6-associated protein (E6-AP) form a complex which binds to p53 and causes its proteolytic degradation [7].

During the different stages of cervical cancer development, there is an accumulation of epigenetic alterations that leads to changes in gene expression [8]. Altered mechanisms of epigenetic regulation in cervical cancer include DNA methylation and post-translational modifications of histone proteins [8]. It has been reported that histone modifying enzymes such as histone deacetylase (HDAC)-1 and HDAC2 are over-expressed in cervical dysplasia and invasive carcinoma [9]. These results suggest that the dysregulation of enzymes that modify histones in cervical cancer are of importance for the biology of this tumor entity.

HR-HPVs establish persistent infection by maintaining their genomes as extrachromosomal elements—the so-called episome—that replicate, together with host DNA, in infected cells [10]. By associating with the host chromatin, HR-HPV redirects the normal cellular control of chromatin to create a cellular environment that is beneficial for both the HR-HPV multiplication and malignant progression of the infected cell. Therefore, the investigation of HPV–host chromatin interaction will offer new insights into the importance of HPV-driven chromatin regulation in cervical cancer tissue [10].

The state of histone modifications that are connected to the early and late HPV viral promoters—modification by acetylation and methylation—were examined in a previous study in cell culture systems using chromatin immunoprecipitation assays: in undifferentiated cells, di-methylated forms of histone H3K4 as well as acetylated histone H3 and H4 were found [11]. Together with differentiation, the levels of di-methylated H3K4 and acetylated H3 are increased, while the acetylated H4 is also increased, which suggests that nucleosomes are activated through histone modifications to coordinate the HPV transcription during cell differentiation [11].

The already-mentioned studies and several other studies showed that histone protein modifications play a fundamental role in HPV driven oncogenesis. Because a systematic investigation of posttranslational changes in histone proteins, for their prognostic relevance in cervical cancer tissue, was lacking, the aim of this study was an expression analyses of histone H3 acetyl K9 (H3K9ac) and histone H3 tri methyl K4 (H3K4me3) in cervical cancer, examined in 250 cases by immunohistochemical methods and assessed by a semi-quantitative score.

## 2. Results

### 2.1. H3K9ac Staining in Cervical Carcinoma

**Figure 1:**
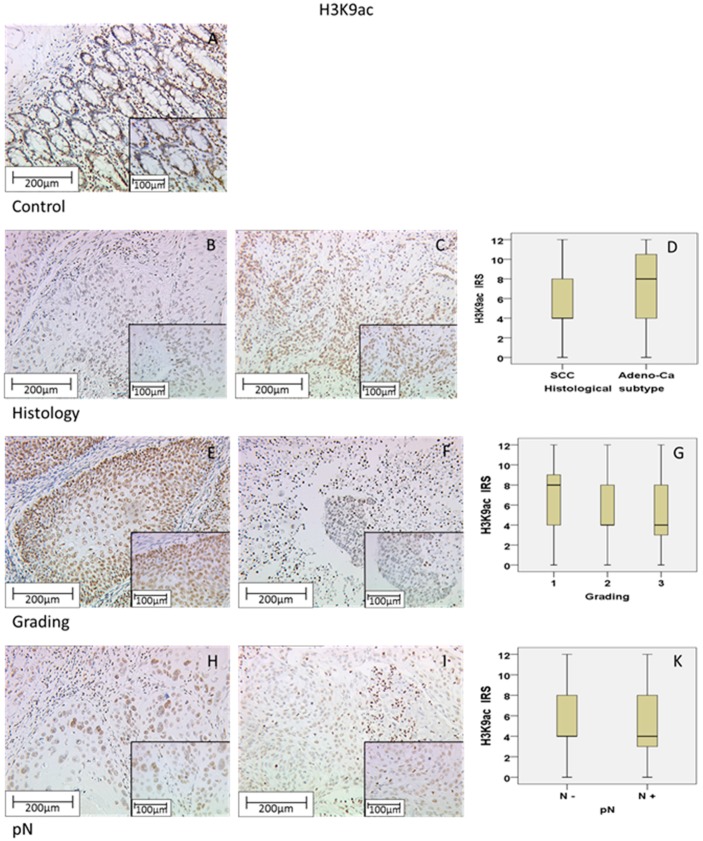

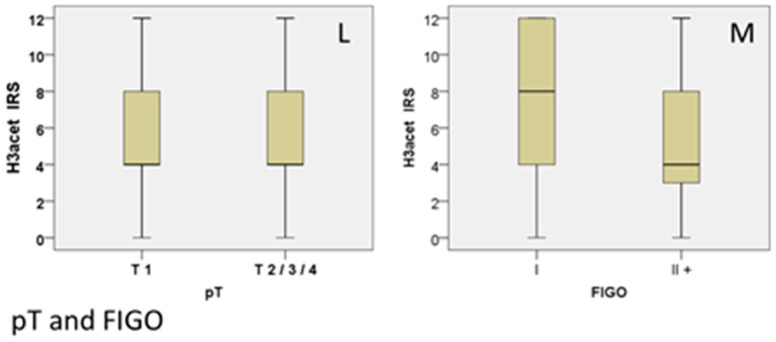
Positive control of H3K9ac staining in colon tissue with strong nuclear cytoplasmic expression and without cytoplasmic expression in epithelial cells (**A**). Squamous epithelial tissue (**B**) showed a median expression of H3K9ac, while adenocarcinoma tissue (**C**) showed significantly more intense H3K9ac staining; the summary regarding histological subtype is shown as a box plot (**D**). Grading: G1-stage tumors showed enhanced H3K9ac expression (**E**), G3-stage tumors (**F**) showed weak staining; the summary regarding grading is shown as a box plot (**G**). N-status: Negative N-status with high H3K9ac expression (**H**), positive N-status with low H3K9ac expression (**I**); the summary regarding N-status is shown as a box plot (**K**). T-status: The median Immune Reactive Score (IRS) is 4 for every T-status (**L**), although there is a strong correlation. TNM classification and the International Federation of Gynecology and Obstetrics (FIGO): Boxplot shows a different median IRS for FIGO-states (**M**). Scale bar 200 µm, small pictures 100 µm.

To control the quality of our H3K9ac staining, we used normal (non-pathological) colon tissue, which showed strong nuclear expression in >80% of epithelial cells without a cytoplasmic expression (Figure 1A).

A total of 92.8% of all cervical cancer specimens showed only a nuclear expression of H3K9ac with a median Immune Reactive Score (IRS) of 4 (36%), while 7.2% of all samples did not express H3K9ac at all. Compared to 50.8% with low expression (IRS = 1–5), an enhanced staining (IRS ≥ 6) was detected in 42.0% of samples.

In the following analyses, we examined the correlation between H3K9ac and several clinic pathological parameters such as histological subtype, grading, T-status, N-status and FIGO-classification by noticing the distribution of these parameters in our study group (Table 1).

Examining the histological subtype, squamous epithelial carcinomas (Figure 1B) with a median IRS of 4 showed a lower H3K9ac expression than adenocarcinoma tissue (Figure 1C) with a median IRS of 8, differing significantly from each other (*p* = 0.013; Figure 1D; Table 2).

Regarding the grading, low graded (G1) specimens did not show the general median IRS of 4 in the H3K9ac staining. They presented a median IRS of 8 in 31% of samples (Figure 1E), while the median IRS of 4 in intermediate graded (G2) and high graded (G3, Figure 1F) samples was represented by 35.0% and 41.0%, respectively. Thus, enhanced staining was highly significantly correlated with low grading (*p* = 0.004; Rho = −0.209 with *p* = 0.001; Figure 1G and Table 2).

Analysing the N-Status (involved lymph nodes), 86.1% of all patients without lymph-node metastasis (N−; Figure 1H) had an IRS of ≥4 compared to 66.0% of all patients with lymph-node positive status (N+; Figure 1I), while both presented the same median IRS of 4 (Figure 1K). An enhanced expression of H3K9ac was accompanied by lymph node-negative status, while low expression was accompanied by lymph node-positive status (*p* = 0.001; Rho = −0.236 with *p* < 0.001; Table 2).

All tumor sizes (T-stages) showed an equal IRS of 4 (Figure 1L), being represented in 34/110 cases (31.0%) in T1-stage patients, and 56/137 cases (40.9%) in T2/3/4-stage patients. Data showed a significant difference (*p* = 0.035) with an inversed correlation meaning that enhanced H3K9ac staining correlated with low T-Status (Rho = −0.149 with *p* = 0.019; Table 2). Although the correlation was highly significant, it was not detectable in the boxplot.

Regarding the FIGO status, patients with FIGO I had a median IRS of 8 in 17 patients in this subgroup (17/64; 26.6%), compared to patients with a FIGO status of II or more with a median IRS of 4 (32/92; 34.8%). We could show a significant correlation between FIGO status and H3acet expression (*p* = 0.016) with a negative spearman’s-rank correlation (Rho = −0.192; *p* = 0.016), meaning that strong H3K9ac staining correlated with low FIGO status (Figure 1M).

In summary, we detected associations of H3K9ac regarding histological subtype (*p* = 0.013), grading (*p* = 0.004), N-status (*p* = 0.001), T-status (*p* = 0.035) and FIGO status (*p* = 0.016) by using non-parametric tests (Table 2). In particular, the negative correlation between H3acet staining on the one hand and FIGO, T- and N-status on the other hand seem to go well together, as FIGO status is defined by T and N-status.

### 2.2. H3K4me3 Staining in Cervical Cancer

**Figure 2:**
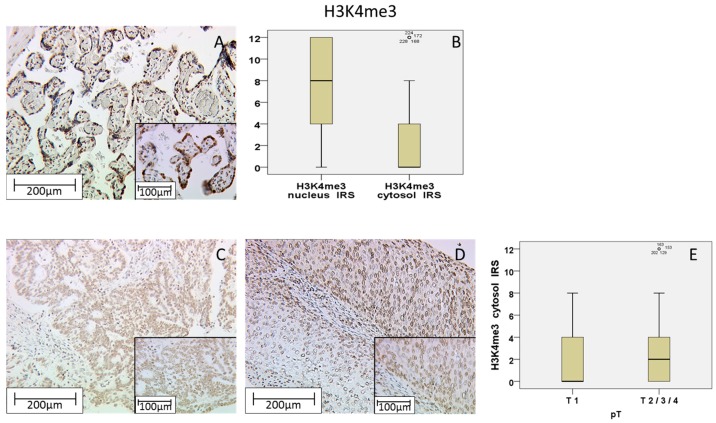
Positive control of H3K4me3 staining in placenta tissue with strong nuclear cytoplasmic expression and weak cytoplasmic expression in trophoblastic cells (**A**); H3K4me3 showed a higher expression in the nucleus than in the cytoplasm (**B**); T1-stage tumors (**C**) with significantly lower expression than T2/3/4-stage tumors (**D**); The summary regarding T-status is shown as a box plot (**E**). Scale bar 200 µm, small pictures 100 µm.

To evaluate the H3K4me3 staining, we used placenta tissue where a very strong expression in trophoblastic cells, in the nucleus as well as in the cytoplasm, was found (Figure 2A).

Of all the cervical cancer specimens, a total of 96.8% showed H3K4me3 expression, while 3.2% did not show any expression at all. H3K4me3 was found in the cytoplasm as well as in the nucleus, correlating significantly with each other (Rho = 0.290 with *p* < 0.001). All positive tested samples presented a nuclear expression with a median IRS of 8 (31%) compared to a median IRS of 0 (56.4%) in samples with cytoplasmic expression (Figure 2B). All in all, nuclear expression of H3K4me3 was detectable in 96.8% (negative: 3.2%) of patients, while cytoplasmic expression was only positive in 43.6% of all patients negative: 56.4%). Nuclear H3K4me3 expression was enhanced (IRS = 4–12) in 88.4% of all cases compared to a low expression (IRS = 0–3) in 11.6%. Regarding cytoplasmic expression, 36.8% slides showed a high expression (IRS = 4–12) and 63.2% slides presented a weak expression (IRS = 0–3).

Examining the T-stage, T1-stage carcinoma tissues showed the general median of 0 in 30.4% of all cases in the cytoplasmic H3K4me3 staining (Figure 2C). In contrast, T2/3/4-stage samples showed an enhanced median IRS of 2 (Figure 2D) in 3.6% of all cases. Performing nonparametric-tests and Spearman’s rank correlation, enhanced cytoplasmic expression of H3K4me3 was correlated with advanced T-Status (*p* = 0.002; Rho = 0.191 with *p* = 0.003; Table 2). This means that an advanced cytoplasmic H3K4me3 expression correlated with higher T-status (Figure 2E).

In summary, we found associations of cytoplasmic H3K4me3 expression regarding T-status (*p* = 0.002) by using non-parametric tests (Table 2). No significant difference was found for cytoplasmic expression among N-Status, FIGO or grading and there were no correlations detected between nuclear H3K4me3 expression and the described pathological parameters.

### 2.3. Correlation Analysis between H3K4me3 and p16 Oncoprotein

It is well known that the expression of p16 oncoprotein in cancer is not only associated with DNA methylation but also with histone modification [12]. By using recently published data by our institute [13,14], we looked for a similar association in cervical cancer. Analyses showed that a nuclear H3K4me3 expression was positively correlated with p16 expression (Rho = 0.144; *p* = 0.027; Table 2). No correlation was found regarding the cytoplasmic expression of H3K4me3 (Rho = 0.009; *p* > 0.05) or nuclear H3K9ac expression (Rho = 0.047; *p* > 0.05).

### 2.4. Role of H3K9ac and H3K4me3 for Overall Survival

Enhanced H3K9ac expression (IRS ≥ 6) was—as well as H3K4me3 expression (IRS ≥ 4)—associated with survival-time after diagnosis.

**Figure 3:**
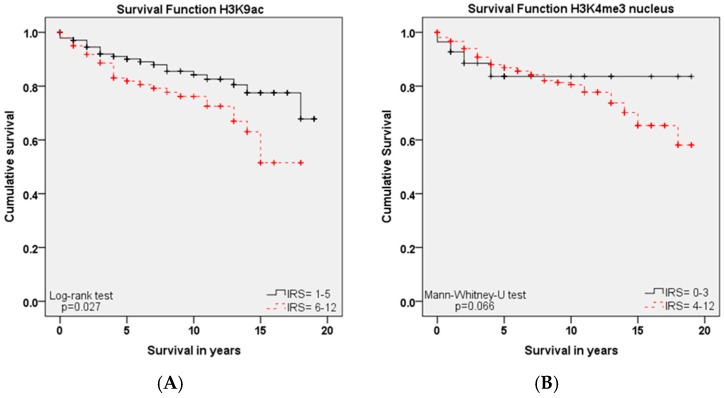
Kaplan–Meier analyses for overall survival: H3K9ac (*p* = 0.027; **A**) with high expression (IRS ≥ 6; red) compared to low expression (IRS ≤ 5; black); High nuclear H3K4me3 expression (IRS ≥ 4; red) compared to low expression (IRS ≤ 3; black) regarding overall survival (*p* = 0.066; **B**).

As shown in the Kaplan–Meier curve (Figure 3A), high expression of H3K9ac (IRS ≥ 6) in cervical cancer patients was correlated with poor prognosis in overall survival rates (*p* = 0.027). This was in contrast to the correlation between H3K9ac staining and the described clinic pathological parameters (T-status, N-status, Grading and FIGO), where high H3K9ac expression was correlated with the low stage of these parameters.

In addition to H3K9ac, we examined the role of H3K4me3 for survival, where we found a similar correlation: advanced nuclear H3K4me3 expression was also correlated with poor prognosis concerning overall survival (Figure 3B, *p* = 0.066). For cytoplasmic H3K4me3 expression, there was no significance concerning overall survival.

### 2.5. Role of H3K9ac and H3K4me3 for Progress-Free Survival

Although H3K9ac and nuclear H3K4me3 expressions showed significant differences regarding overall survival, their expressions showed no significant correlation for relapse-free survival (*p* = 0.763 and *p* = 0.08).

**Figure 4:**
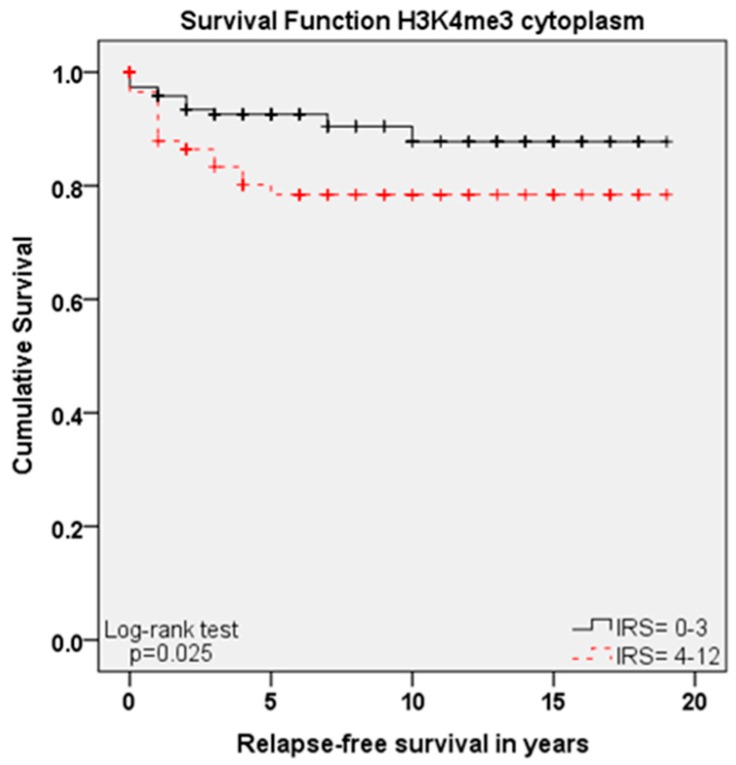
Kaplan–Meier analyses for relapse-free survival: high cytoplasmic H3K4me3 expression (IRS ≥ 4; red) compared to low expression (IRS ≤ 3; black) regarding relapse-free survival (*p* = 0.025).

In contrast, cytoplasmic H3K4me3 expression, which was not a marker of overall survival, was significantly correlated with progress-free survival: high cytoplasmic expression of H3K4me3 (IRS ≥ 4) meant short relapse-free survival (Figure 4, *p* = 0.025), matching the correlation between high expression and advanced T-Status.

### 2.6. Cox Regression of H3K9ac and H3K4me3 and Clinic Pathological Variables

The additionally performed multivariate cox-regression tested which histopathological parameters were independent prognosticators for survival in our study-group.

For overall survival, the histological subtype (*p* = 0.040), pN-status (*p* = 0.003), FIGO classification (*p* = 0.012), age at surgery (*p* < 0.001) and the expression of H3K9ac (*p* = 0.027) were independent prognosticators, but not the H3MK4me3 expression or other tested clinic pathological parameters (Table 3).

Regarding relapse-free survival, only FIGO status (*p* = 0.044) and cytoplasmic H3K4me3 expression (*p* = 0.030) turned out to be independent markers in multivariate cox-analysis (Table 4), but not H3K9ac expression, nuclear H3K4me3 expression or other described parameters.

## 3. Discussion

Within this study, we showed that the immunohistochemical evaluation of histone H3K9ac staining was correlated with low grading, low FIGO-classification, low T-status and negative N-status in cervical cancer. We could also find a higher expression of histone H3K9ac in adenocarcinoma compared to squamous cell carcinoma. Due to its correlation between expression and poor prognosis (overall survival), it could be used as an independent marker of prognosis. Cytoplasmic expression of histone H3K4me3 in a cervical cancer specimen was correlated with advanced T-status and poor prognosis. It seems to be a marker of relapse-free survival, while nuclear expression showed a correlation to poor prognosis without being an independent marker regarding cervical cancer.

Histone proteins give the genome the ability to pack very large amounts of DNA in a very small space, but at the same time they leave their N-terminal tails flexible [15]. The N-terminal tail of the histone proteins can undergo post-translational modification by enzymes, adding chemical modifications such as acetylation, methylation, phosphorylation and deamination that alter the structure of the DNA package and allow or prevent gene transcription [16]. It is already known that histone modifications at histone 3 lysine 9 acetylation (H3K9ac) denote active regions at enhancers as well as promoters, whereas the tri-methyl form, H3K4me3, preferentially identifies the gene promoters that are active [17,18]. In addition, it has been shown that epigenetic modulations of the genome involve histone modifications that alter the gene chromatin configuration. A decondensed (“open”) configuration allows transcription factors access to binding sites, whereas a condensed (“closed”) configuration blocks transcription binding sites, thereby regulating gene transcription [19,20]. Based on these findings, it has been shown that high metastatic potential had greater acetylation of histone H3 lysine 9 (H3K9ac) and tri-methylation of histone H3 lysine 4 (H3K4me3) [19]. Therefore, these two modifications that are correlated to enhanced gene activity and in addition show high metastatic potential were used in the present study as markers for the identification of the prognostic relevance of those histone modifications for cervical cancer survival.

It is already known that E6 oncoprotein and E6-associated protein (E6-AP) form a complex which binds to p53 and causes its proteolytic degradation [7]. P53 is a tumor suppressor, as it leads to cell cycle arrest or apoptosis in the case of DNA damage [21]. As E6 oncoprotein induces the degradation of p53, the function of this important cell cycle protein is disturbed [12] after HPV infection. The cell cycle regulation protein p16 is expressed at high levels in HPV-infected epithelial cells, which is why it acts as a marker for the diagnosis of a HPV associated carcinoma [22,23]. On the other hand, studies have shown that p16 expression is induced by an oncogene senescence-related mechanism that involves histone H3K27 demethylation by histone lysine demethylase, and that p16 expression is necessary for the survival of HPV-infected cells expressing E7 viral oncoprotein [24,25].

Unfortunately, p16 is not exclusively increased by E7 oncoprotein in carcinogenesis. Therefore, in a recent study, we established and published an immunohistochemical approach for the direct detection of E6 oncoprotein in uterine cervical cancer [14]. In addition, we found a very high mutation rate of TP53 in this cancer type where p53 is initially inactivated via E6 during the development of cervical cancer. An unexpected finding is the correlation of this mutation with better survival, possibly due to better response to therapy [13].

Because both H3K9ac and H3K4me3 are negative prognosticators for cervical cancer patients, the use of epigenetic drugs or the search for epigenetic targets could be a useful goal for cervical cancer treatment.

Recently, two main classes of epigenetic drugs—methylation inhibitors and HDAC inhibitors—are in clinical trials for the treatment of cervical cancer [26]. One of these potential new drugs could be valproic acid (VPA). VPA was found to be an effective inhibitor of histone deacetylases and has been shown to induce anti-tumor effects by modulating cellular pathways, including cell cycle arrest, apoptosis, angiogenesis, metastasis, differentiation, and senescence [27]. The antitumor effect of VPA in cervical cancer can be explained by either the hyper-acetylation of p53 protein, protecting it from degradation by E6 and increasing p53 activity; or via the inhibition of Akt1 and Akt2 expression, which results in apoptotic cell death [28,29]. Acetylation of p53 is a process that occurs in response to DNA damage and stress and is necessary for p53 transcriptional activity. Therefore, p53 was one of the first non-histone proteins that could be acetylated by histone acetyl transferases [30].

In addition, HDAC inhibitors also interfere with cervical cancer via non-histone targets. The HDAC inhibitor suberoylanilide hydroxamic acid (SAHA) induces apoptosis in HeLa cervical cancer cells in vitro with bortezomib by activating caspase-3 and increasing the ratio of bax/bcl-2 expression [31]. Epigenetic aberrations, such as histone protein modification have the ability to regulate the expression of oncogenes or repression of tumor suppressor genes. Therefore, these modified histone proteins are powerful candidates for the investigation of cancer pathogenesis and progression. For cervical cancer, for instance, Feng et al. [26] highlighted a number of genes that underwent epigenetic alteration at the level of DNA methylation, histone modification, or noncoding RNA action in this type of cancer.

Further investigation of these alterations and information about them could lead to new and reliable screening methods for women at high risk of cervical cancer and can help to establish new candidates for a better treatment of this disease.

## 4. Materials and Methods

### 4.1. Patients and Specimens

We used 250 paraffin-embedded cervical cancer samples obtained from patients having undergone surgery for cervical cancer in the Department of Obstetrics and Gynecology of the Ludwig-Maximilians-University of Munich between 1993 and 2002. The median age of the patients was 47.0 years (range 20–83 years), and overall median survival was 100.0 months. For distribution of clinic pathological variables see Table 1. Only patients with adenocarcinoma or squamous cell carcinoma (SCC) of the cervix were included in our study, other histological subtypes were excluded due to low number. As positive controls for immunohistochemically staining, we utilized colon tissue for histone H3 acetyl K9 and placenta tissue for histone H3 tri methyl K4; both received from the Department of Obstetrics and Gynecology of the Ludwig-Maximilians-University of Munich. Clinical and follow-up data for statistical analyses were provided by the Munich cancer registry and retrieved from medical records.

### 4.2. Ethics Approval

All cervical cancer specimens had originally been collected for histopathological diagnostics and were no longer used for clinical tests, when they were recruited for this survey. Patient data were totally anonymized and the authors were blinded for clinical information—including survival-time during experimental analyses. The study was conducted conforming to the Declaration of Helsinki and was approved by the local ethics committee of the Ludwig-Maximilians University of Munich (reference number 259-16, 2016).

### 4.3. Immunohistochemistry

The paraffin-embedded and formalin-fixed samples (3 µm) had been stored at room temperature and were first dewaxed in xylol. After rinsing the tissue in 100% ethanol and blocking the endogenous peroxidase with 3% methanol/H_2_O_2_, the samples were rehydrated in a descending alcohol series. To avoid heat-associated protein-agglomeration and to unmask the antigen, the slides were warmed up to 100 °C in a pressure cooker for 5 min, adding a trisodium citrate buffer solution with pH = 6. After having prepared the slides by washing them in distilled water and PBS-buffer, we added the suitable blocking solution to avoid unspecific (hydrophobic) bindings between immunoglobulins on the one side and cell membranes or fatty tissue on the other side by saturation of electrostatic charges. Afterwards, the samples were incubated at a temperature of +4 °C with the primary antibodies (Table 5).

After increasing the staining by the post-block-reagent and applying the HRP-polymer, the substrate-staining with DAB was performed and the counterstaining by haemalm (2 min) was carried out subsequently. More details concerning the suitable detection system and the following steps were defined exactly in Table 5. Finally, the samples were dehydrogenated in a rising alcohol series and covered. Colon tissue and placenta tissue were used for each staining as positive and negative controls for H3K9ac and H3K4me3.

The intensity of the expression was evaluated by the immunoreactive score (IRS). Well-established and applied in numerous other studies, this semi-quantitative score multiplies the intensity of the staining (0 = not stained; 1 = low intensity; 2 = moderate intensity; 3 = high intensity) and the percentage of stained cells (0 = 0%; 1 = 1%–10%; 2 = 11%–50%; 3 = 51%–80%; 4 ≥ 80%). Finally, we distinguished between 0 = no expression and 12 = very high expression of histones.

### 4.4. Statistics

For statistical analyses, IBM SPSS Statistics version 23 (Armonk, NY, USA) was used. Bivariate correlations were calculated by Spearman’s-rank-correlation coefficient and non-parametric tests (NPAR: Mann–Whitney U test, Kruskal–Wallis test) were employed to compare independent groups. To visualize differences concerning survival rates, Kaplan–Meier curves were created and afterwards compared by a log-rank test and—if necessary—Mann–Whitney-U test. Survival times are shown in years, but for more exact results they were calculated in months. To show statistical difference, *p* had to be <0.05.

## 5. Conclusions

The expression of histone H3 acetyl K9 and histone H3 tri methyl K4 was examined in 250 cases of cervical cancer. Both histone protein modifications turned out to be independent negative prognosticators for the overall survival or the relapse-free survival of cervical cancer patients. For cervical cancer, it is the first study that showed a direct link between histone protein modification and survival in a large cohort of patients.

## Figures and Tables

**Table 1:** Clinic pathological variables of the patients included in this study.

Item	No./Total No.	%
**Age, years**		
<49	139/250	55.6
>49	111/250	44.4
**Number of Positive Nodes**		
0	151/250	60.4
≥1	97/250	38.8
Not available (NA’s)	2/250	0.8
**Tumor Size, pT**		
pT1	110250	44
pT2/3/4	137/250	54.8
Not available (NA’s)	3/250	1.2
**FIGO**		
I	64/250	25.6
II/III/IV	92/250	36.8
Not available (NA’s)	94/250	37.6
**Tumor Grade**		
I	21/250	8.4
II	143/250	57.2
III	78/250	31.2
Not available (NA’s)	8/250	3.2
**Tumor Subtype**		
Squamous	202/250	80.8
Adenocarcinoma	48/250	19.2
**Progression** (over 235 months)		
None	210/250	84
At least one	21/250	11.6
Not available (NA’s)	11/250	4.4
**Survival** (over 235 months)		
Right censured	190/250	76
Died	49/250	19.6
Not available (NA’s)	11/250	4.4

**Table 2:** Staining results and correlation analysis.

	H3K9ac				H3K4me3 (Cytoplasmic)				H3K4me3 (Nuclear)			
	Median IRS (+/−SD)	%	*p* (NPAR)	ρ	Median IRS (+/−SD)	%	*p* (NPAR)	ρ	Median IRS (+/−SD)	%	*p* (NPAR)	ρ
**Histology**												
**SCC**	4 (+/−3.45)	40.10%	0.013	-	0 (+/−2.67)	57.40%	0.296	-	8 (+/−3.56)	32.20%	0.603	-
**Adeno-Ca**	8 (+/−3.78)	31.30%			0 (+/−3.35)	52.10%			8 (+/−3.58)	27.10%		
**Grade**												
**G1**	8 (+/−3.51)	31.00%	0.004	−0.209 (*p* = 0.001)	0 (+/−2.94)	57.10%	0.197	0.082 (*p* > 0.05)	8 (+/−3.66)	28.60%	0.917	−0.017 (*p* > 0.05)
**G2**	4 (+/−3.57)	35.00%			0 (+/−3.08)	52.40%			8 (+/−3.59)	31.50%		
**G3**	4 (+/−3.26)	41.00%			0 (+/−2.24)	62.80%			8 (+/−3.55)	33.30%		
**pN**												
**N−**	4 (+/−3.55)	86.10%	0.001	−0.236 (*p* = 0.000)	0 (+/−3.02)	57.00%	0.981	−0.001 (*p* > 0.05)	8 (+/−3.50)	32.50%	0.695	0.025 (*p* > 0.05)
**N+**	4 (+/−3.33)	66.00%			0 (+/−2.43)	55.70%			8 (+/−3.69)	28.90%		
**pT**												
**T1**	4 (+/−3.52)	30.90%	0.035	−0.149 (*p* = 0.019)	0 (+/−2.34)	69.10%	0.002	0.191 (*p* = 0.003)	8 (+/−3.49)	30.00%	0.171	0.081 (*p* > 0.05)
**T2/3/4**	4 (+/−3.49)	40.90%			2 (+/−2.93)	3.60%			8 (+/−3.60)	32.10%		
**FIGO**												
**I**	8 (+/−3.91)	26.60%	0.016	−0.192 (*p* = 0.016)	0 (+/−2.45)	64.10%	0.324	0.070 (*p* = 0.384)	8 (+/−3.37)	32.80%	0.862	−0.005 (*p* = 0.948)
**II+**	4 (+/−3.44)	23.90%			2 (+/−2.66)	5.40%			8 (+/−3.67)	30.40%		
**p16**	-	-	-	0.047 (*p* > 0.05)	-	-	-	0.009 (*p* > 0.05)	-	-	-	0.144 (*p* = 0.027)

SD = standard deviation; % = percentage of the subgroup with median IRS; NPAR = non-parametric test; *p* = *p*-value; ρ = correlation coefficient; SCC = squamous cell carcinoma, pT = tumor size, FIGO = TNM classification and the International Federation of Gynecology and Obstetrics.

**Table 3:** Cox regression of clinic pathological variables regarding overall survival.

Variable	Significance	Hazard Ratio of Exp(B)	Lower 95% CI of Exp(B)	Upper 95% CI of Exp(B)
Histology	0.040	1.893	1.030	3.479
pT	0.751	0.910	0.509	1.626
pN	0.003	2.447	1.367	4.380
FIGO	0.012	3.181	1.287	7.863
Grading	0.198	1.360	0.852	2.170
Age at surgery	0.000	1.049	1.026	1.072
H3K9ac	0.027	1.900	1.076	3.356
H3K4me3 nucleus	0.708	1.216	0.436	3.389
H3K4me3 cytoplasm	0.159	1.503	0.853	2.651

**Table 4:** Co12x regression of clinic pathological variables regarding progress-free survival.

Variable	Significance	Hazard Ratio of Exp(B)	Lower 95% CI of Exp(B)	Upper 95% CI of Exp(B)
Histology	0.753	1.156	0.469	2.851
pT	0.760	0.890	0.423	1.875
pN	0.843	1.082	0.495	2.368
FIGO	0.044	3.085	1.031	9.235
Grading	0.521	1.228	0.656	2.299
Age at surgery	0.157	1.021	0.992	1.052
H3K9ac	0.763	0.890	0.417	1.900
H3K4me3 nucleus	0.476	0.681	0.236	1.963
H3K4me3 cytoplasm	0.030	2.278	1.084	4.790

**Table 5:** Antibodies and chemicals used for the immunohistochemistry.

Histone H3 Acetyl K9 ^1^	Histone H3 Tri Methyl K4 ^2^
Blocking solution ^3^: 5 min	Blocking solution ^3^: 5 min
primary antibody ^1^: 1:200 in PBS ^5^, incubation: 16 h, 4 °C	primary antibody ^2^: 1:500 in PBS ^5^, incubation: 16 h, 4 °C
PostBlock ^3^: 20 min	PostBlock ^3^: 20 min
HRP Polymer ^3^: 30 min	HRP Polymer ^3^: 30 min
Chromogen: DAB ^4^ (0.5 min)	Chromogen: DAB ^4^ (1 min)

^1^ Anti Histone H3 acetyl K9, clone Y28 (rabbit IgG), concentration: 0.059 mg/mL, company: Abcam (Cambridge, UK), order number: ab32129; ^2^ Anti Histone H3 tri methyl K4, rabbit IgG polyclonal, concentration: 1 mg/mL, company: Abcam, order number: ab8580; ^3^ ZytoChem Plus HRP Polymer Kit (Mouse/Rabbit) 3 × 100; company: Zytomed Systems (Berlin, Germany) Nr. POLHRP-100; ^4^ Liquid DAB + Substrate Chromogen System 1 mg/mL, DAKO; ^5^ Dulbecco’s Phosphate Buffered Saline.

## Abbreviations

H3K9ac	histone H3 acetyl K9
H3K4me3	histone H3 tri methyl K4
SCC	squamous cell carcinoma
